# Bilingual Aphasia Test in Urdu and its Clinical Implications

**DOI:** 10.12669/pjms.37.5.4572

**Published:** 2021

**Authors:** Nadir Ali, Muhammad Shaban Rafi

**Affiliations:** 1 Dr. Nadir Ali, MD. Department of Linguistics and Communications, Institute of Liberal Arts University of Management and Technology, Lahore, Pakistan; 2 Dr. Muhammad Shaban Rafi, PhD. Department of Linguistics and Communications, Institute of Liberal Arts University of Management and Technology, Lahore, Pakistan

Dear Editor,

A language is core to express identity, thoughts, and feelings. Aphasia alters language performance on all levels of linguistic skills.^1^ It is essential to mention the significance of language assessment of persons who have lost their language skills due to aphasia in Pakistan, where a majority of the population is bilingual.

Bilingual aphasia is a frequent phenomenon in clinical settings. People practice different languages in different communicative contexts. The language used at work may be different from that at home or in their social sphere. In a bilingual context, all languages collectively contribute to everyday life. To evaluate the real impact of aphasia in routine life, assessing the linguistic deficit in each language is necessary. The effectiveness of treatment intervention depends upon the administration of a valid and reliable test.^2^

In bilingual aphasics, symptoms can be varied and could encompass all the languages that he/she speaks. It is necessary to assess the patient’s linguistic ability with each of the languages that was spoken in his/her premorbid circumstances with the help of a cross-linguistically equivalent test. Therefore, its assessment and treatment may become more challenging as compared to monolingual aphasics. Thus, in a progressively bilingual or multilingual Pakistan, the impact of stroke on such individuals’ communication abilities is tremendous.^3^

Most developed and validated aphasia batteries are in the English language (e.g., Boston Diagnostic Aphasia Examination and Western Aphasia Battery). In Pakistan, the options left for clinicians for assessment of language in bilingual aphasics is to either use institutionally-organized, non-standardized translations of batteries originally constructed for native English speakers, considering their specific socio-cultural and linguistic context, or to use the Urdu version of the Bilingual Aphasia Test (BAT-Urdu), which is a structural and cultural adaptation to the idiosyncrasies of the Urdu language. However, BAT-Urdu has not been standardized and normed on patients with aphasia. Hence, the test has not been clinically validated in the bilingual population by obtaining psychometric data (either not obtained or published in English). Consequently, it prevents easy access for the larger audience of linguistic aphasiology researchers from evaluating the reliability and validity of the test.

BAT, which is considered an equivalent test to assess the differential linguistic impairment in bilinguals, is adapted culturally and linguistically to many languages. It is the most-used clinical tool and research instrument for assessing residual linguistic performance in aphasics, comprised of three parts. Part-A of the test evaluates the bilingual background of the patients and their families. Part-B measures their performance in different modalities of language through various comprehension and expression tasks. Part-C examines the patient’s ability to translate the content of one of his/her languages to another. Despite its general flexibility, its invalidity in the Pakistani context hinders the reliability of the diagnosis.^4^

Considering the lack of clinical data and the unavailability of a valid assessment test for Urdu/Punjabi speaking aphasics, not only adaptation of BAT for each of the bilingual contexts but also validation of the culturally and linguistically adapted test is a pressing issue, requiring attention and resolution. This humble effort can highlight the clinical implications of this significant subject to the knowledge of academia through your reputable journal. With the prevalently limited context of diagnosis of bilingual aphasia in mind, it will ultimately help clinicians, practitioners, and speech-language pathologists in their diagnostic assessment of linguistic deficits to restore lost ability to communicate.

## Authors’ Contribution

**NA** conceived, designed, drafted and editing of manuscript.

**MSR** did critical review and final approval of manuscript.
